# Molecular analysis of the interaction between ubiquitin-specific protease 7 and large T antigen of Merkel cell polyomavirus

**DOI:** 10.71150/jm.2511009

**Published:** 2026-02-12

**Authors:** Dahwan Lim, Jung-Hwan Park, Ho-Chul Shin, Seung Jun Kim, Bonsu Ku

**Affiliations:** 1 Orphan Disease Therapeutic Target Research Center, Korea Research Institute of Bioscience and Biotechnology, Daejeon 34141, Republic of Korea; 2 Infectious Diseases Therapeutic Research Center, Korea Research Institute of Chemical Technology, Daejeon 34114, Republic of Korea; 3 Division of Gastroenterology, Department of Internal Medicine, Yonsei University College of Medicine, Seoul 03722, Republic of Korea; 4 Critical Diseases Diagnostics Convergence Research Center, Korea Research Institute of Bioscience and Biotechnology, Daejeon 34141, Republic of Korea

**Keywords:** ubiquitin-specific protease 7, Usp7, large T antigen, LT, Merkel cell polyomavirus, MCPyV

## Abstract

Merkel cell polyomavirus (MCPyV) is the primary causative agent of Merkel cell carcinoma, a rare but highly aggressive neuroendocrine skin cancer. Large T antigen (LT), one of two oncoproteins encoded by MCPyV, sustains the proliferation of MCPyV-infected tumor cells. LT contains multiple protein-binding motifs that mediate interactions with diverse host proteins essential for its function. Among these, ubiquitin-specific protease 7 (Usp7), a deubiquitinase that regulates the stability of multiple substrates, including p53, is a recently identified LT-interacting protein. In the present study, we characterized the intermolecular interaction between Usp7 and MCPyV LT using biochemical analyses and AlphaFold-based structural modeling. Our results demonstrate that MCPyV LT directly interacts with the TRAF domain of Usp7 via a unique binding motif that is distinct from the canonical sequence. Moreover, MCPyV LT attenuates the p53-deubiquitinating activity of Usp7, providing insights into the molecular function of this viral oncoprotein.

## Introduction

Merkel cell polyomavirus (MCPyV) is a non-enveloped virus with a double-stranded circular DNA genome encapsidated in an icosahedral capsid (Ahmed et al., 2021; Houben et al., 2023). MCPyV belongs to the *Alphapolyomavirus* genus of the *Polyomaviridae* family, members of which infect a wide range of vertebrate hosts. MCPyV is the causative agent of approximately 80% of cases of Merkel cell carcinoma, a rare but highly aggressive neuroendocrine skin cancer, and is therefore classified as one of seven recognized human oncoviruses (Feng et al., 2008; Liu et al., 2016). The MCPyV genome consists of three regions: a late region encoding two structural viral capsid proteins (viral proteins 1 and 2), an early region encoding T antigens, and a non-coding region (Pietropaolo et al., 2020). Alternative splicing of the early region produces four transcripts: large T antigen (LT), small T antigen (ST), 57k T antigen, and alternative frame of the large T open reading frame (ALTO). Among these, LT and ST are viral oncoproteins involved in the proliferation of MCPyV-infected tumor cells through the regulation of viral and cellular transcription, viral DNA replication, host cell cycle progression, and suppression of immune responses (Houben et al., 2023). In contrast, ALTO functions as a tumor suppressor by downregulating viral replication and supporting persistent infection (Salisbury et al., 2024; Wang et al., 2024). The precise functional role of 57k T antigen remains unclear.

**Fig. 1.**
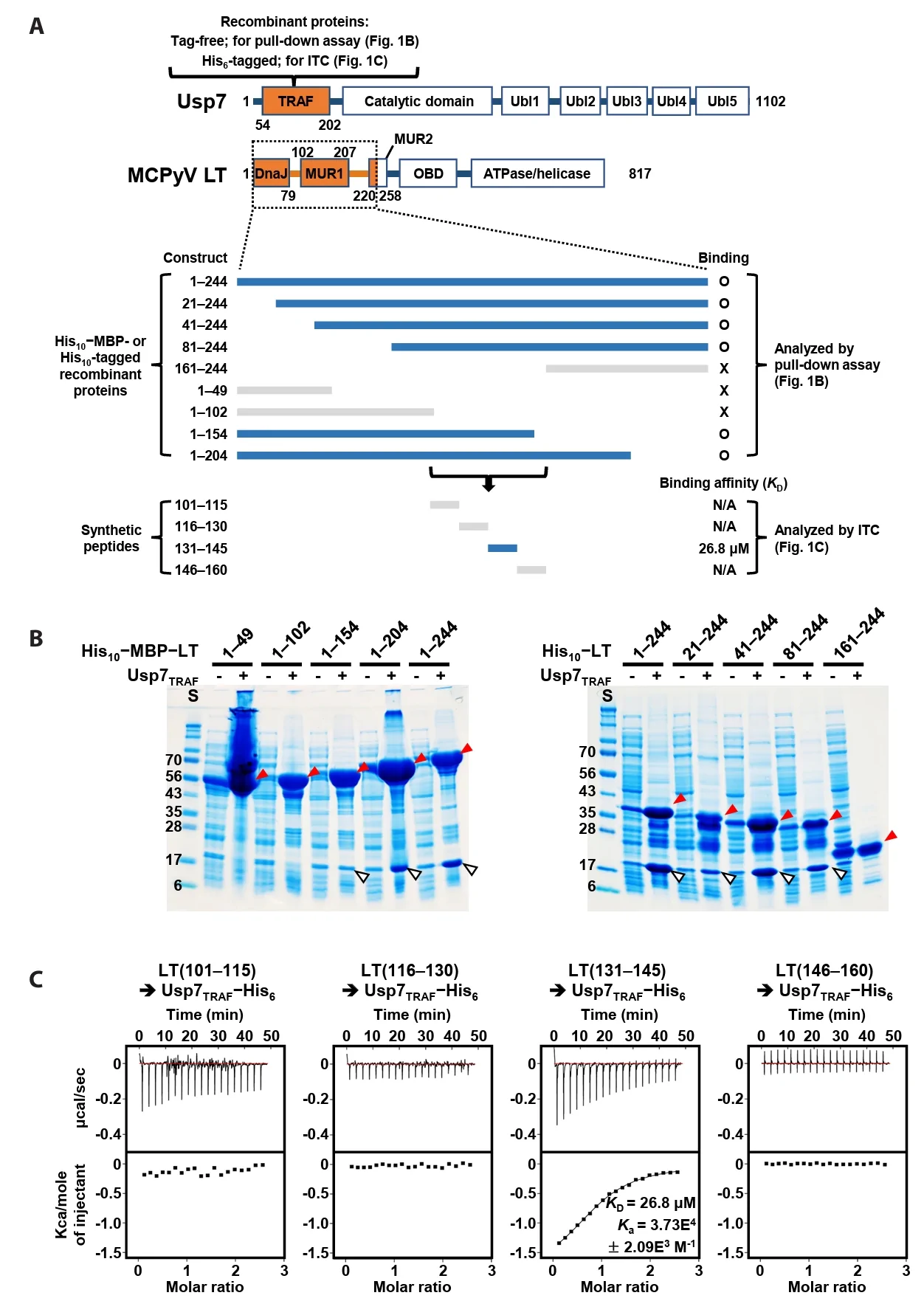
Verification of direct interaction between Usp7_TRAF_ and MCPyV LT. (A) Schematic representation of the Usp7 and MCPyV LT constructs used for binding analyses. The two initial constructs are indicated in orange at the top. Thirteen truncated MCPyV LT constructs tested by pull-down assay and ITC are shown at the bottom. LT constructs that bound to Usp7_TRAF_ are highlighted in navy, whereas non-binding constructs are shown in gray. (B) Pull-down assay. Interactions between tag-free Usp7_TRAF_ and the 10 indicated (His)_10_−MBP-tagged (left) or (His)_10_-tagged (right) MCPyV LT constructs were examined using Ni-NTA chromatography. Eluted fractions were visualized using SDS–PAGE. Red and white arrowheads denote (His)_10_−MBP-tagged or (His)_10_-tagged MCPyV LT and tag-free Usp7_TRAF_ proteins, respectively. S, size marker. (C) ITC analysis. Each peptide (1 mM) was titrated into 100 μM recombinant Usp7 as indicated. *K*_D_ and *K*_a_ values were deduced from the curve fitting of the integrated heat per mole of added ligand.

MCPyV LT is composed of several functional domains and host protein-binding motifs (Fig. 1A). The N-terminal DnaJ domain, which contains a heat shock cognate protein 70-interacting motif, promotes viral replication (Kwun et al., 2009; Pietropaolo et al., 2020). The subsequent Merkel cell polyomavirus unique region (MUR) domain, which harbors a vacuolar morphogenesis protein 6-binding motif, regulates LT stability (Borchert et al., 2014; Nwogu et al., 2020; Pietropaolo et al., 2020). The MUR domain is divided into two parts: the region that precedes the retinoblastoma protein-binding LxCxE motif is MUR1, while the region that follows the motif is MUR2. The central origin-binding domain, which recognizes the MCPyV origin of replication, and C-terminal helicase/ATPase domain are essential for viral genome replication (Harrison et al., 2011; Pietropaolo et al., 2020). These functional domains and host-interacting motifs are key to the function of MCPyV LT as the major regulator of MCPyV replication and pathogenic processes.

Ubiquitin-specific protease 7 (Usp7) is a deubiquitinase that modulates the stability of multiple substrates, including p53, and plays critical roles in immune regulation, DNA damage repair, and viral infection (El-Hamaky et al., 2025). It has also emerged as a promising therapeutic target, as Usp7 is frequently overexpressed in various tumors and in viral infections (El-Hamaky et al., 2025; Song et al., 2025). The N-terminal tumor necrosis factor receptor-associated (TRAF) domain of Usp7 recruits several proteins, including the human proteins p53, MDM2, MDM4, MCMBP, and UbE2E1 (Jagannathan et al., 2014; Sarkari et al., 2010, 2013; Sheng et al., 2006), and the viral proteins vIRF1 and vIRF4 from Kaposi’s sarcoma-associated herpesvirus (KSHV) and EBNA1 from Epstein–Barr virus (EBV) (Chavoshi et al., 2016; Lee et al., 2011; Saridakis et al., 2005). The “P/A/E−x−x−S” motif commonly found in these proteins is responsible for binding to the TRAF domain of Usp7. Recently, Czech-Sioli et al. (2020) reported that MCPyV LT coprecipitates with both endogenous and bacterially expressed Usp7 and that this interaction requires the TRAF domain of Usp7. They also attempted to identify the TRAF domain-binding region in MCPyV LT, and noted the presence of several conventional “P/A/E−x−x−S” motifs as candidates (Czech-Sioli et al., 2020). Furthermore, their study showed that MCPyV LT is not deubiquitinated by Usp7, but instead competes with p53 for Usp7 binding (Czech-Sioli et al., 2020). Here, we analyzed the interaction between MCPyV LT and Usp7, which led to the identification of a unique TRAF domain-binding motif within MCPyV LT. We also demonstrated that MCPyV LT attenuated the p53-deubiquitinating activity of Usp7, providing important insight into the functional role of this viral–host interaction.

## Materials and Methods

### Preparation of recombinant proteins

The DNA fragment encoding residues 54–207 of human Usp7 was subcloned into a pET21a plasmid (Novagen, USA) to produce either a tag-free or a C-terminal (His)_6_-tagged recombinant protein. The (His)_6_-tagged construct was used as a template to generate the D102A, D102E, and D102L mutant Usp7 proteins. The DNA fragment encoding residues 1–244 of MCPyV LT was subcloned into modified pET21a plasmids (Novagen) to produce recombinant proteins fused to either a (His)_10_−maltose-binding protein (MBP) tag or a (His)_10_ tag. MCPyV LT fragments comprising residues 21–244, 41–244, 81–244, 161–244, 1–49, 1–102, 1–154, and 1–204 were generated using the two constructs encoding residues 1–244 as templates. Proteins were expressed in *Escherichia coli* BL21(DE3) RIL cells (Novagen) grown in Luria–Bertani medium at 25°C for 16 h, after induction with 0.5 mM isopropyl β-D-thiogalactopyranoside. The resulting cell lysates were used for pull-down assays and isothermal titration calorimetry (ITC) measurements.

### Pull-down assays

Cell lysates expressing (His)_10_−MBP-tagged MCPyV LT or tag-free Usp7 were mixed and used for Ni–NTA affinity chromatography (QIAGEN, Germany). The samples were washed sequentially with a buffer containing 50 mM Tris-HCl (pH 7.5) and 200 mM NaCl, followed by washing with buffers supplemented with 30 and 40 mM imidazole. The final samples were eluted using buffer supplemented with 200 mM imidazole and 3 mM β-mercaptoethanol. For visualization, protein samples were separated by sodium dodecyl sulfate–polyacrylamide gel electrophoresis (SDS–PAGE) and stained with Coomassie Brilliant Blue.

### ITC measurements

Recombinant proteins used for ITC analysis were purified using Ni-NTA affinity chromatography and a HiLoad 26/600 Superdex 75 prep grade size-exclusion chromatography column (Cytiva, USA), followed by equilibration in buffer containing 50 mM Tris-HCl (pH 7.5), 200 mM NaCl, and 3 mM β-mercaptoethanol. Synthetic peptides derived from MCPyV LT, including residues 101–115, 116–130, 131–145 (wild-type; P135D; S138A; P135D·S138A), 145–160, 131–139, 134–145, 139–145 (wild-type; pS142), were purchased from Dandicure (Korea) and then dialyzed against the final buffer used for Usp7 purification. ITC was performed using 100 μM recombinant Usp7 proteins and 1 mM synthetic MCPyV LT peptides as previously described (Jung et al., 2025; Lim et al., 2021).

### Immunoprecipitation assay

The p53 C-His clone was provided by the Korea Human Gene Bank, Medical Genomics Research Center (Korea; clone ID: KE1338_mCHis). The pRK5-HA-ubiquitin-WT plasmid was gifted by Prof. Ted Dawson (Addgene plasmid #17608). Myc−Usp7, Flag−LT, and Flag−LT (139–145) were subcloned into the pcDNA3.1 vector. Human embryonic kidney 293T (HEK293T) cells cultured in 100-mm dishes were transfected with each plasmid using Lipofectamine 3000 according to the manufacturer’s instructions. For immunoprecipitation, 500 μg of total cell lysate was incubated with antibodies and TruBlot protein G magnetic beads (Rockland Immunochemicals, Inc., USA) overnight at 4℃ with gentle rocking. The beads were then washed and subjected to SDS–PAGE. For immunoblot analysis, 60 μg of each total cell lysate and immunoprecipitation sample was resolved by SDS–PAGE, transferred to membranes, and probed with antibodies. Chemiluminescent signals were detected using SuperSignal West Pico Chemiluminescent Substrate (Pierce Biotechnology, Inc., USA) and visualized using a Fusion Solo X imaging system (Vilber Lourmat, France).

## Results

### Identification of Usp7-binding region in MCPyV LT

A previous study by Czech-Sioli et al. (2020) reported that the N-terminal region of MCPyV LT regulates viral genome replication by associating with the TRAF domain of Usp7 (Usp7_TRAF_ in this study). To characterize this interaction more precisely, we performed binding analyses using recombinant proteins produced in *E. coli* and synthetic peptides. First, we expressed tag-free Usp7_TRAF_ and a series of truncated LT constructs tagged with (His)_10_-linked MBP or (His)_10_-tag (Fig. 1A) and examined their interactions in pull-down assays using Ni-NTA affinity chromatography. As shown in Fig. 1B, Usp7_TRAF_ bound to MCPyV LT constructs comprising residues 1–244, 21–244, 41–244, 81–244, 1–204, and 1–154, but not to those spanning residues 1–49, 1–102, and 161–244. These results indicated that the Usp7_TRAF_-binding motif is located within residues 101–160 of MCPyV LT. In the full-length structural model of MCPyV LT predicted by AlphaFold 3 (https://alphafoldserver.com), this region adopts an unstructured loop conformation and is thus likely exposed and accessible for protein binding (Fig. S1). To identify the minimal region required for binding to Usp7_TRAF_, we synthesized four 15-mer peptides encompassing residues 101–160 of MCPyV LT and examined their interactions with Usp7_TRAF_ using ITC. Among these, only the MCPyV LT(131–145) peptide interacted with Usp7_TRAF_ with a dissociation constant (*K*_D_) of 26.8 μM (Fig. 1C). Taken together, these results demonstrate that residues 131–145 of MCPyV LT, part of the MUR domain, harbor the motif essential for the interaction with Usp7_TRAF_.

### MCPyV LT harbors a distinct Usp7_TRAF_-binding motif

**Fig. 2.**
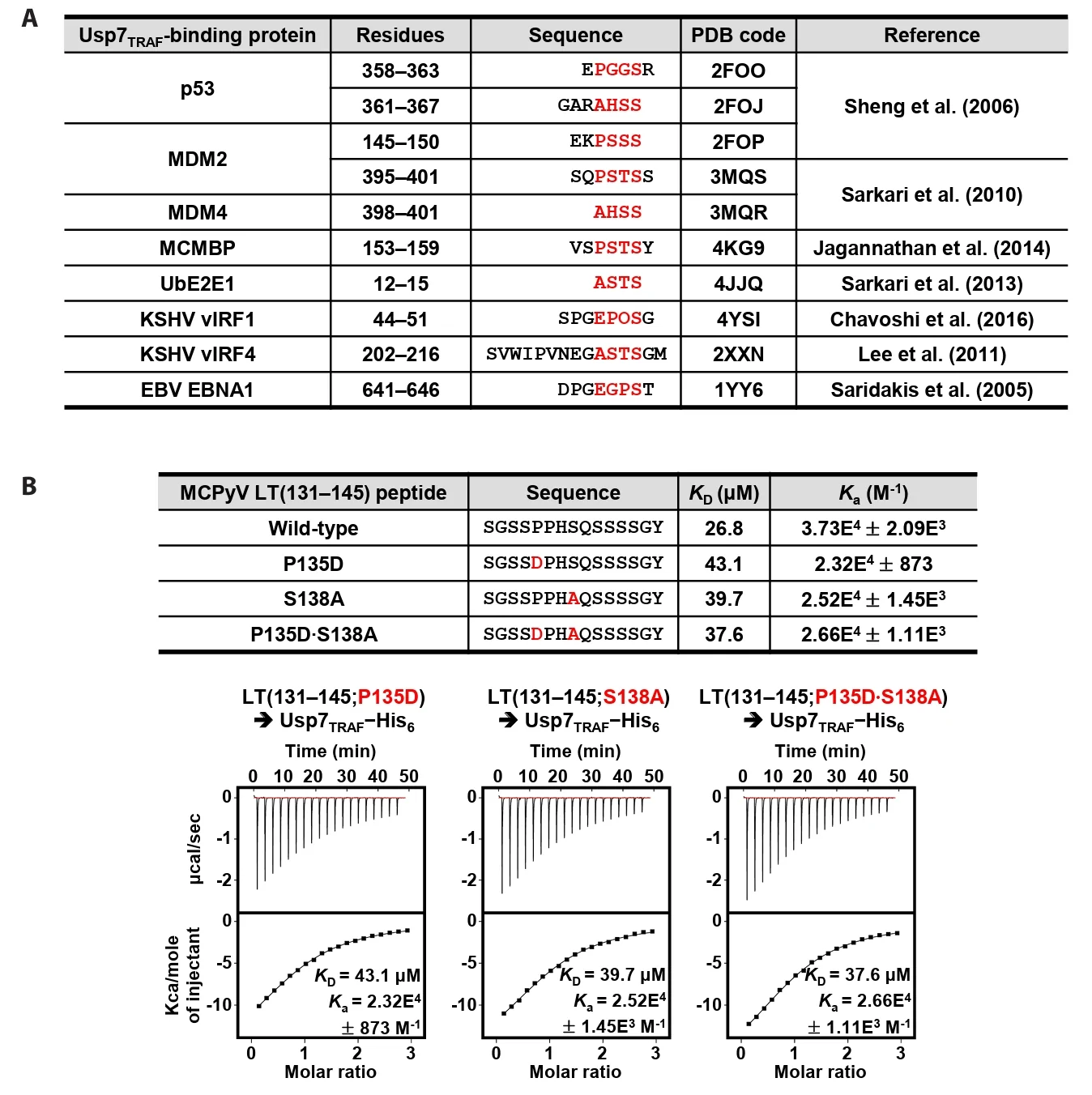
MCPyV LT does not require the canonical motif for its interaction with Usp7_TRAF_. (A) Usp7_TRAF_-binding proteins are shown together with their residue numbers, amino acid sequences, PDB codes, and references. P/A/E−x−x−S motif residues are highlighted in red. (B) Interaction between Usp7_TRAF_ and three mutant MCPyV LT(131–145) peptides was assessed using ITC. Mutated residues are highlighted in red.

**Fig. 3.**
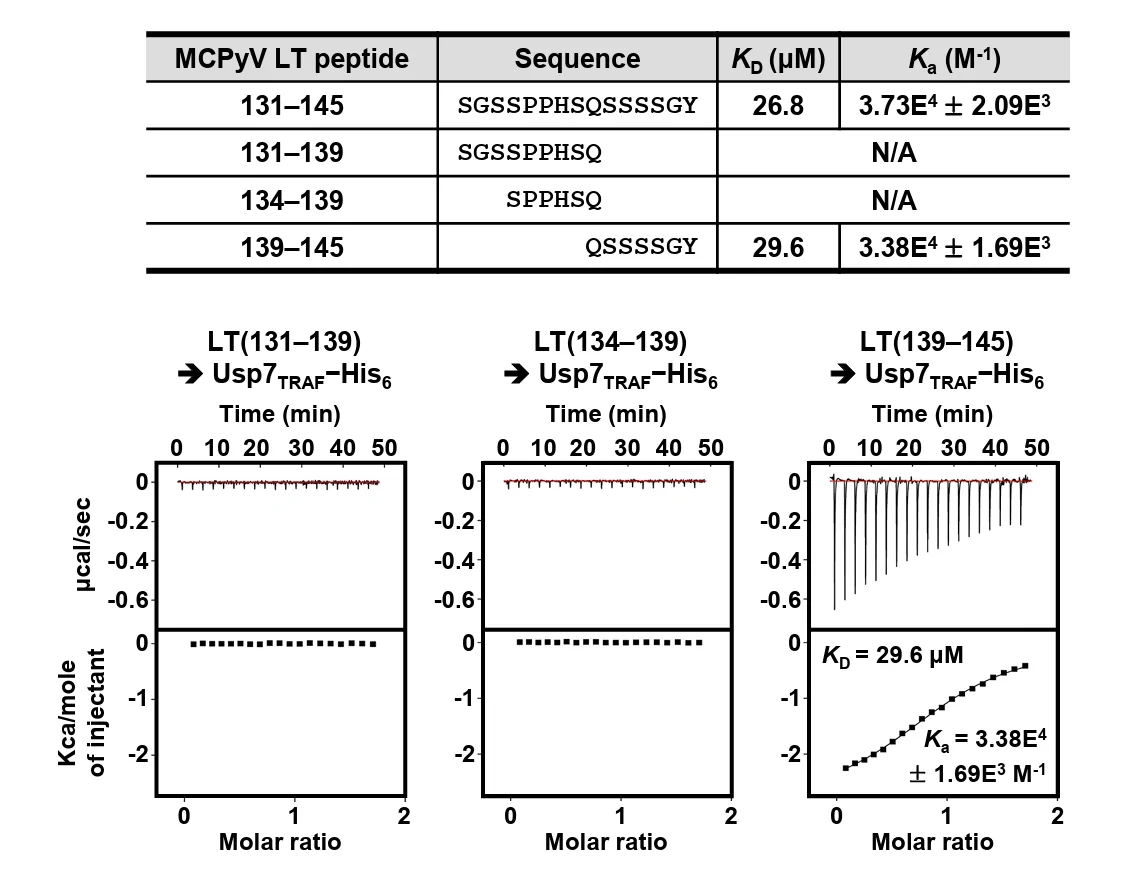
Mapping of Usp7_TRAF_-binding motif in MCPyV LT. Interaction between Usp7_TRAF_ and three indicated MCPyV LT peptides was assessed using ITC.

Residues 131–145 of MCPyV LT contain Pro^135^−Pro−His−Ser^138^ sequences, which correspond to the Usp7_TRAF_-binding motif (Fig. 2A). Thus, we hypothesized that this region mediates the interaction with Usp7_TRAF_. Unexpectedly, the P135D and/or Ser138A substitutions in MCPyV LT did not impair binding to Usp7_TRAF_ (Fig. 2B). Furthermore, neither the MCPyV LT(131–139) nor LT(134–139) peptide showed a detectable association with Usp7_TRAF_ in ITC analysis (Fig. 3), demonstrating that this interaction is not mediated by the Pro^135^−Pro−His−Ser^138^ motif. In contrast, the interaction with Usp7_TRAF_ was detected in an ITC experiment using the MCPyV LT(139–145; Gln^139^−Ser−Ser−Ser−Ser−Gly−Tyr^145^) peptide with a *K*_D_ of 29.6 μM, comparable to that of the MCPyV LT(131–145) peptide (*K*_D_ of 26.8 μM) (Fig. 3). Collectively, these results demonstrate that MCPyV LT possesses a noncanonical Usp7_TRAF_-binding motif distinct from those found in other Usp7-binding proteins.

### AlphaFold-based modeling of the Usp7_TRAF_−MCPyV LT complex

**Fig. 4.**
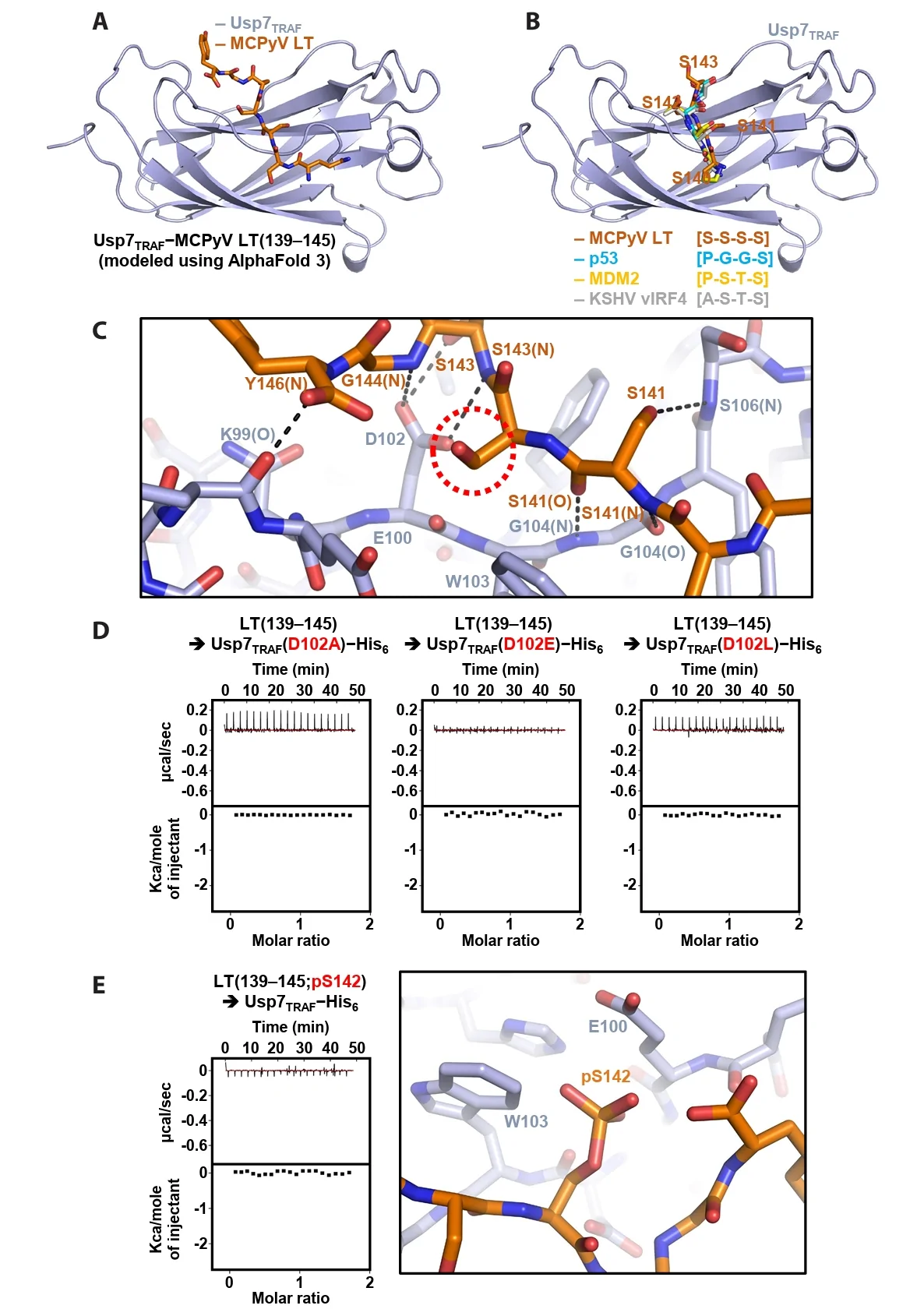
Modeling and verification of the Usp7_TRAF_−MCPyV LT complex. (A) Structural model of Usp7_TRAF_ in complex with MCPyV LT (residues 139–145) generated using AlphaFold 3. (B) Superposition of the Usp7_TRAF_−MCPyV LT model with the crystal structures of Usp7_TRAF_ bound to p53 (PDB code: 2F1X), MDM2 (PDB code: 3MQS), or KSHV vIRF4 (PDB code: 2XXN). For clarity, only MCPyV LT-bound Usp7_TRAF_ and four residues of Usp7_TRAF_-bound fragments corresponding to residues 140–143 of MCPyV LT are shown. (C) Intermolecular interactions between Usp7_TRAF_ and MCPyV LT in the AlphaFold 3-based model. Hydrogen bonds are shown as dashed lines, and interaction-involved residues are labeled. N and O in parentheses denote main chain amide and carbonyl groups, respectively. A red dashed circle highlights the side chain of Ser142, whose phosphorylation effect was examined in (E). (D) Effects of Asp102 substitution in Usp7_TRAF_ on binding interactions with the MCPyV LT(139–145) peptide were analyzed using ITC. Mutations are highlighted in red. (E) Effects of Ser142 phosphorylation in the MCPyV LT(139–145) peptide on binding interactions were analyzed using ITC (left) and modeled by AlphaFold 3 (right).

Next, the Usp7_TRAF_−MCPyV LT(139–145) complex was modeled using AlphaFold 3 (Fig. 4A). In the structural model, Usp7_TRAF_ adopts an antiparallel β-sandwich fold, creating a shallow surface groove that accommodates MCPyV LT(139–145) (Fig. 4A). Comparison with previously determined crystal structures of Usp7_TRAF_ bound to the p53-, MDM2- or KSHV vIRF4-derived motifs (Hu et al., 2006; Lee et al., 2011; Sarkari et al., 2010) showed that the P/A/E−x−x−S motif in these structures corresponds to the serine stretch (Ser^140^−Ser−Ser−Ser^143^) in MCPyV LT(139–145) (Fig. 4B). Based on this model, the intermolecular interactions between Usp7_TRAF_ and MCPyV LT were analyzed. Hydrogen bonding appears to be a major determinant of this interaction. These bonds involve the side chain hydroxyl groups of Ser141 and Ser143 in MCPyV LT; side chain carboxyl group of Asp102 in Usp7_TRAF_; main chain amide groups of Ser141, Ser143, Gly144, and Tyr146 in MCPyV LT and Gly104 and Ser106 in Usp7_TRAF_; and main chain carbonyl groups of Ser141 in MCPyV LT and Lys99 and Gly104 in Usp7_TRAF_ (Fig. 4C).

In the modeling analysis, the side chain carboxyl group of Asp102 in Usp7_TRAF_ was found to play a critical role in the formation of multiple hydrogen bonds with the main chain amides of Ser143 and Ser144 and side chain hydroxyl group of Ser143 in MCPyV LT (Fig. 4C). Thus, three Usp7_TRAF_ mutants were generated by substituting Asp102 with alanine, glutamate, or leucine, and their binding to the MCPyV LT(139–145) peptide was examined using ITC. As shown in Fig. 4D, none of the mutants exhibited detectable binding, verifying the essential role of Asp102 in Usp7_TRAF_ in the interaction with MCPyV LT. Next, we prepared the MCPyV LT(139–145; pS142) peptide phosphorylated at Ser142, as this modification is thought to be required for recruiting β-transducin repeat-containing protein (β-TrCP), an E3 ligase, and promoting subsequent degradation of MCPyV LT (Nwogu et al., 2020; Pham and Kwun, 2024). ITC analysis revealed that this peptide did not interact with Usp7_TRAF_ (Fig. 4E, left), likely because of steric hindrance and electrical repulsion between phosphorylated Ser142 in MCPyV LT and Glu100 and Trp103 in Usp7_TRAF_ (Fig. 4E, right). These findings suggest that phosphorylation of Ser142 serves as a switch in MCPyV LT, promoting binding to the E3 ubiquitin ligase β-TrCP while preventing association with the deubiquitinating enzyme Usp7.

### MCPyV LT suppresses the deubiquitinating activity of Usp7

**Fig. 5.**
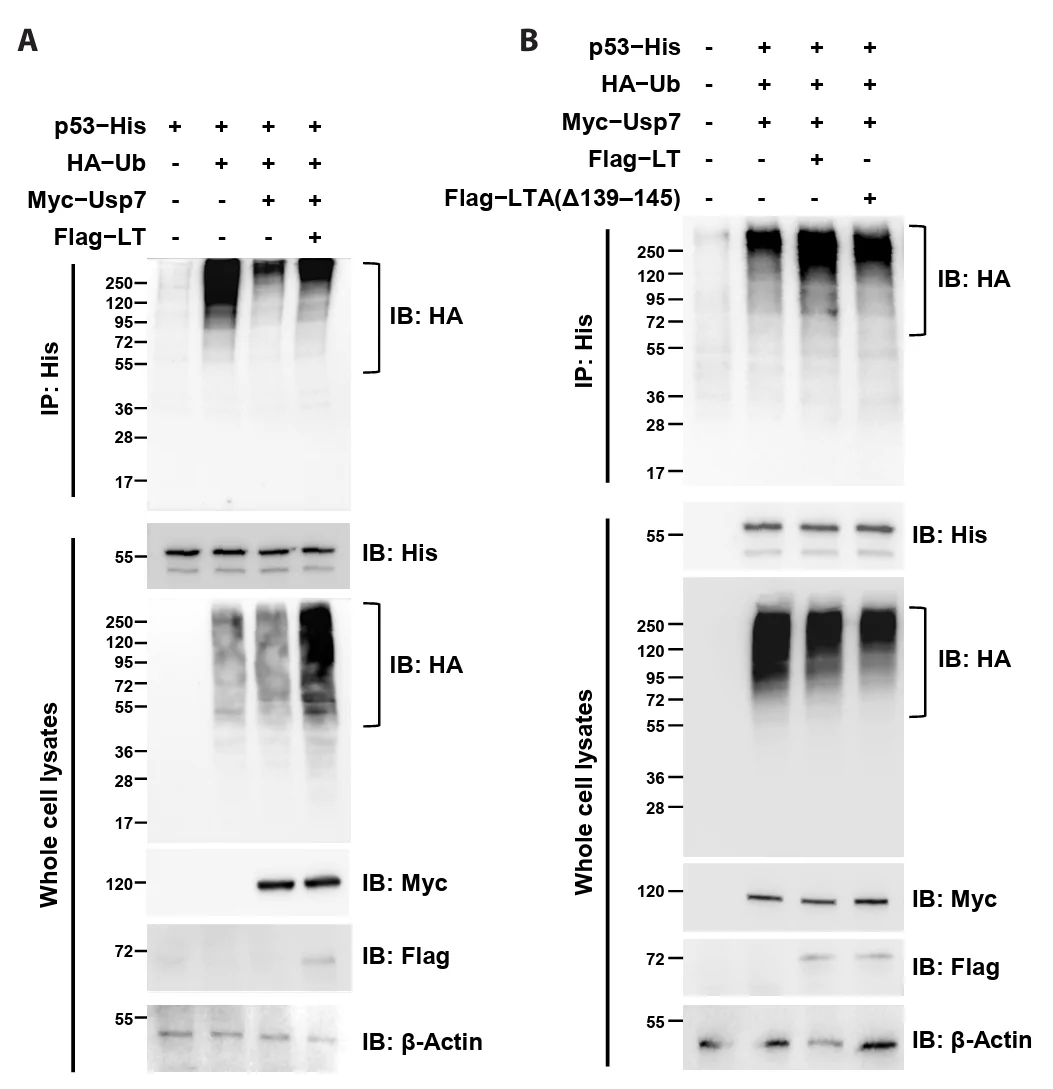
MCPyV LT attenuates the p53 deubiquitination activity of Usp7. Co-immunoprecipitation analysis. The extent of p53 ubiquitination in HEK293T cells transiently expressing the indicated proteins was examined using immunoprecipitation and immunoblotting. The deubiquitination activity of Usp7 was attenuated by co-expression of MCPyV LT (A) but not by the MCPyV LT mutant lacking residues 139–145 (B). β-Actin was used as a loading control. IP, immunoprecipitation; IB, immunoblotting.

Because our data demonstrated that MCPyV LT directly interacts with Usp7_TRAF_, we hypothesized that LT interferes with the deubiquitinating activity of Usp7. To evaluate this hypothesis, constructs expressing Flag-tagged MCPyV LT, Myc-tagged Usp7, HA-tagged ubiquitin, and His-tagged p53 (used as a ubiquitination substrate) were generated, and the extent of p53 ubiquitination was analyzed using immunoprecipitation. In HEK293T cells, p53 ubiquitination accumulated when p53 was co-expressed with ubiquitin (Fig. 5A, lanes 1 and 2) but was markedly decreased when Usp7 was also expressed (Fig. 5A, lane 3). Strikingly, co-expression with MCPyV LT restored ubiquitinated p53 levels (Fig. 5A, lane 4; Fig. 5B, lanes 2 and 3), indicating that this viral protein antagonized Usp7-mediated deubiquitination. In contrast, deletion of residues 139–145 in MCPyV LT impaired this activity (Fig. 5B, lane 4). Taken together, these results strongly suggest that MCPyV LT attenuates the p53-deubiquitinating activity of Usp7 through its interaction with Usp7_TRAF_.

## Discussion

In the present study, we investigated the direct interaction between Usp7_TRAF_ and MCPyV LT using multiple biochemical approaches. Our results demonstrated that Usp7_TRAF_ directly interacts with MCPyV LT (Fig. 1), and further identified the precise Usp7_TRAF_-binding motif in the MUR domain of MCPyV LT (Figs. 2 and 3). As the Usp7_TRAF_-binding motif of MCPyV LT is exposed and does not appear to require a conformational change for binding (Fig. S1), the affinity of Usp7_TRAF_ for the MCPyV LT(139–145) peptide (*K*_D_ of 29.6 μM; Fig. 3) is expected to be comparable to that for the full-length protein. Notably, this motif, comprising residues 139–145 (Gln^139^−Ser−Ser−Ser−Ser−Gly−Tyr^145^), is distinct from the canonical P/A/E−x−x−S motif. Although Czech-Sioli et al. (2020) previously suggested that MCPyV LT uses either a p53/MDM2-like P/A−x−x−S sequence or an EBNA1-like E−x−x−S sequence, our data, further supported by AlphaFold-mediated structural modeling (Fig. 4), indicate that MCPyV LT employs an unexpected motif to associate with Usp7_TRAF_. These results also indicate that both host and viral proteins harbor additional unidentified motifs that enable interactions with Usp7_TRAF_, which warrants further investigation.

MCPyV is a member of the *Polyomaviridae* family, which includes simian virus 40, BK polyomavirus, and John Cunningham polyomavirus. All these viruses express LT proteins that commonly harbor the DnaJ, OBD, and helicase/ATPase domains (Googins et al., 2025). However, the MUR domain (residues 102–258) and Usp7_TRAF_-binding motif (Gln^139^−Ser−Ser−Ser−Ser−Gly−Tyr^145^) are present only in MCPyV, implying that targeting Usp7_TRAF_ represents a unique strategy of this virus. Interestingly, unlike the LT proteins of simian virus 40, BK polyomavirus, and John Cunningham polyomavirus, which directly engage p53 and suppress its apoptosis-inducing activity (Baez et al., 2017; Lilyestrom et al., 2006; Shivakumar and Das, 1996; Staib et al., 1996), MCPyV LT does not bind p53 (Borchert et al., 2014; Cheng et al., 2013). Therefore, we hypothesized that interaction with Usp7 represents an alternative mechanism by which MCPyV LT regulates the activity of p53, an established substrate of this deubiquitinase. MCPyV LT was previously reported not to be a substrate of Usp7 but to compete with p53 for Usp7 binding (Czech-Sioli et al., 2020). Consistently, our structural and biochemical analyses demonstrated that MCPyV LT not only competes with p53 for binding to Usp7_TRAF_ (Fig. 4B), but also attenuates the cellular p53-deubiquitinating activity of Usp7 (Fig. 5). These results suggest that MCPyV LT promotes p53 ubiquitination by interacting with Usp7 and displacing p53 from this deubiquitinase, which should be further investigated. Taken together, our findings provide a strong basis for future studies to determine the precise function of MCPyV LT in MCPyV infection-dependent Merkel cell carcinoma.

## Supplementary Information

Fig. S1. AlphaFold 3-predicted full-length structural model of MCPyV LT. Residues 101–160 of MCPyV LT are shown in marine, while the remaining residues are shown in orange. The side chains of the USP7TRAF-bindinn motif of MCPyV LT, comprising residues 139–145, are represented as sticks and indicated. The three well-folded domains of MCPyV LT, DnaJ, OBD, and ATPase/helicase, are labeled.

## References

[b1-jm-2511009] Ahmed MM, Cushman CH, DeCaprio JA (2021). Merkel cell polyomavirus: oncogenesis in a stable genome. Viruses.

[b2-jm-2511009] Baez CF, Brandao Varella R, Villani S, Delbue S (2017). Human polyomaviruses: the battle of large and small tumor antigens. Virology.

[b3-jm-2511009] Borchert S, Czech-Sioli M, Neumann F, Schmidt C, Wimmer P (2014). High-affinity Rb binding, p53 inhibition, subcellular localization, and transformation by wild-type or tumor-derived shortened Merkel cell polyomavirus large T antigens. J Virol.

[b4-jm-2511009] Chavoshi S, Egorova O, Lacdao IK, Farhadi S, Sheng Y (2016). Identification of Kaposi sarcoma herpesvirus (KSHV) vIRF1 protein as a novel interaction partner of human deubiquitinase USP7. J Biol Chem.

[b5-jm-2511009] Cheng J, Rozenblatt-Rosen O, Paulson KG, Nghiem P, DeCaprio JA (2013). Merkel cell polyomavirus large T antigen has growth-promoting and inhibitory activities. J Virol.

[b6-jm-2511009] Czech-Sioli M, Siebels S, Radau S, Zahedi RP, Schmidt C (2020). The ubiquitin-specific protease Usp7, a novel Merkel cell polyomavirus large T-antigen interaction partner, modulates viral DNA replication. J Virol.

[b7-jm-2511009] El-Hamaky AA, El-Hamamsy MH, El-Moselhy TF, Sharafeldin N, Tawfik HO (2025). Therapeutic targeting of ubiquitin-specific protease 7 (USP7): mechanistic insights, dysregulation, and advances in drug discovery. Eur J Med Chem.

[b8-jm-2511009] Feng H, Shuda M, Chang Y, Moore PS (2008). Clonal integration of a polyomavirus in human Merkel cell carcinoma. Science.

[b9-jm-2511009] Googins MR, An P, Gauthier CH, Pipas JM (2025). Polyomavirus large T antigens: unraveling a complex interactome. Tumour Virus Res.

[b10-jm-2511009] Harrison CJ, Meinke G, Kwun HJ, Rogalin H, Phelan PJ (2011). Asymmetric assembly of Merkel cell polyomavirus large T-antigen origin binding domains at the viral origin. J Mol Biol.

[b11-jm-2511009] Houben R, Celikdemir B, Kervarrec T, Schrama D (2023). Merkel cell polyomavirus: infection, genome, transcripts and its role in development of Merkel cell carcinoma. Cancers.

[b12-jm-2511009] Hu M, Gu L, Li M, Jeffrey PD, Gu W (2006). Structural basis of competitive recognition of p53 and MDM2 by HAUSP/USP7: implications for the regulation of the p53-MDM2 pathway. PLoS Biol.

[b13-jm-2511009] Jagannathan M, Nguyen T, Gallo D, Luthra N, Brown GW (2014). A role for USP7 in DNA replication. Mol Cell Biol.

[b14-jm-2511009] Jung S, Lim D, Choi JS, Shin HC, Kim SJ (2025). Crystal structures of the μ2 subunit of clathrin-adaptor protein 2 in complex with peptides derived from human papillomavirus 16 E7. J Microbiol.

[b15-jm-2511009] Kwun HJ, Guastafierro A, Shuda M, Meinke G, Bohm A (2009). The minimum replication origin of Merkel cell polyomavirus has a unique large T-antigen loading architecture and requires small T-antigen expression for optimal replication. J Virol.

[b16-jm-2511009] Lee HR, Choi WC, Lee S, Hwang J, Hwang E (2011). Bilateral inhibition of HAUSP deubiquitinase by a viral interferon regulatory factor protein. Nat Struct Mol Biol.

[b17-jm-2511009] Lilyestrom W, Klein MG, Zhang R, Joachimiak A, Chen XS (2006). Crystal structure of SV40 large T-antigen bound to p53: interplay between a viral oncoprotein and a cellular tumor suppressor. Genes Dev.

[b18-jm-2511009] Lim D, Shin HC, Choi JS, Kim SJ, Ku B (2021). Crystal structure of human LC8 bound to a peptide from Ebola virus VP35. J Microbiol.

[b19-jm-2511009] Liu W, MacDonald M, You J (2016). Merkel cell polyomavirus infection and Merkel cell carcinoma. Curr Opin Virol.

[b20-jm-2511009] Nwogu N, Ortiz LE, Kwun HJ (2020). Merkel cell polyomavirus large T antigen unique domain regulates its own protein stability and cell growth. Viruses.

[b21-jm-2511009] Pham AM, Kwun HJ (2024). Casein kinase 1α mediates phosphorylation of the Merkel cell polyomavirus large T antigen for β-TrCP destruction complex interaction and subsequent degradation. mBio.

[b22-jm-2511009] Pietropaolo V, Prezioso C, Moens U (2020). Merkel cell polyomavirus and Merkel cell carcinoma. Cancers.

[b23-jm-2511009] Salisbury NJH, Amonkar S, Landazuri Vinueza J, Carter JJ, Roman A (2024). Polyomavirus ALTOs, but not MTs, downregulate viral early gene expression by activating the NF-κB pathway. Proc Natl Acad Sci USA.

[b24-jm-2511009] Saridakis V, Sheng Y, Sarkari F, Holowaty MN, Shire K (2005). Structure of the p53-binding domain of HAUSP/USP7 bound to Epstein-Barr nuclear antigen 1: implications for EBV-mediated immortalization. Mol Cell.

[b25-jm-2511009] Sarkari F, La Delfa A, Arrowsmith CH, Frappier L, Sheng Y (2010). Further insight into substrate recognition by USP7: structural and biochemical analysis of the HdmX and Hdm2 interactions with USP7. J Mol Biol.

[b26-jm-2511009] Sarkari F, Wheaton K, La Delfa A, Mohamed M, Shaikh F (2013). Ubiquitin-specific protease 7 is a regulator of ubiquitin-conjugating enzyme UbE2E1. J Biol Chem.

[b27-jm-2511009] Sheng Y, Saridakis V, Sarkari F, Duan S, Wu T (2006). Molecular recognition of p53 and MDM2 by USP7/HAUSP. Nat Struct Mol Biol.

[b28-jm-2511009] Shivakumar CV, Das GC (1996). Interaction of human polyomavirus BK with the tumor-suppressor protein p53. Oncogene.

[b29-jm-2511009] Song Y, Ren X, Xiong J, Wang W, Zhao Q (2025). Ubiquitin-specific protease 7 (USP7) as a promising therapeutic target for drug discovery: from mechanisms to therapies. J Med Chem.

[b30-jm-2511009] Staib C, Pesch J, Gerwig R, Gerber JK, Brehm U (1996). p53 inhibits JC virus DNA replication in vivo and interacts with JC virus large T-antigen. Virology.

[b31-jm-2511009] Wang R, Senay TE, Luo TT, Liu W, Regan JM (2024). Merkel cell polyomavirus protein ALTO modulates TBK1 activity to support persistent infection. PLoS Pathog.

