# Supplementary material for: Molecular analysis of the interaction between ubiquitin-specific protease 7 and large T antigen of Merkel cell polyomavirus
Source: J Microbiol. 2026 Feb 12;64(2):e2511009. doi: 10.71150/jm.2511009 (PMC13577026; doi:10.71150/jm.2511009)
Supplement: Supplementary file 1 [file jm-2511009-Supplementary-Fig-S1.pdf]

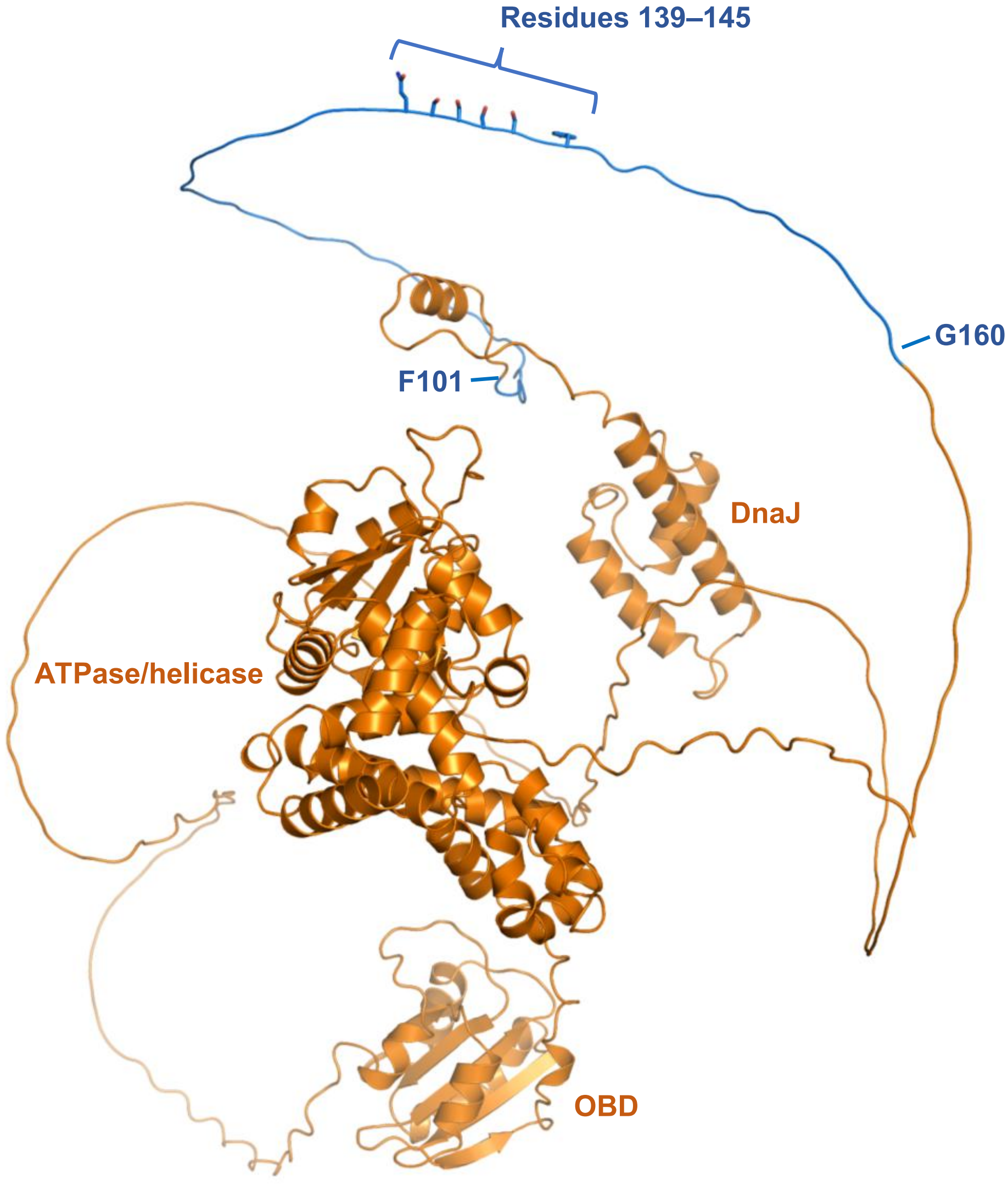


Fig. S1. AlphaFold 3-predicted full-length structural model of MCPyV LT. Residues 101–160 of MCPyV LT are shown in marine, while the remaining residues are shown in orange. The side chains of the USP7TRAF-bindinn motif of MCPyV LT, comprising residues 139–145, are represented as sticks and indicated. The three well-folded domains of MCPyV LT, DnaJ, OBD, and ATPase/helicase, are labeled.
